# LDL-C: An Important Independent Risk Factor for New-Onset Heart Block in Patients with Severe Aortic Stenosis and Heart Failure after TAVR

**DOI:** 10.31083/j.rcm2408243

**Published:** 2023-08-24

**Authors:** Mei Dong, Lizhen Wang, Gary Tse, Tao Dai, Tonglian Lv, Nan Zhang, Lihong Wang, Zhicheng Xiao, Tienan Chen, Tong Liu, Faxin Ren

**Affiliations:** ^1^Department of Cardiology, Affiliated Yantai Yuhuangding Hospital of Qingdao University, 264000 Yantai, Shandong, China; ^2^Tianjin Key Laboratory of Ionic-Molecular Function of Cardiovascular Disease, Department of Cardiology, Tianjin Institute of Cardiology, Second Hospital of Tianjin Medical University, 300070 Tianjin, China; ^3^School of Nursing and Health Studies, Hong Kong, Metropolitan University, 999077 Hong Kong, China; ^4^Kent and Medway Medical School, CT2 7FS Canterbury, UK; ^5^Department of Ultrasound, Affiliated Yantai Yuhuangding Hospital of Qingdao University, 264000 Yantai, Shandong, China; ^6^Department of Cardiovascular Surgery, Second Hospital of Tianjin Medical University, 300070 Tianjin, China

**Keywords:** heart block, heart failure, risk factors, severe aortic stenosis, transcatheter aortic valve replacement

## Abstract

**Background:**

Transcatheter aortic valve replacement (TAVR) is an effective alternative treatment for patients with aortic stenosis (AS) who have intermediate to high surgical risk or who are inoperable. However, the incidence of conduction abnormalities is high after TAVR, which can reduce the effectiveness of the surgery. Our research objective is to explore the risk factors of new-onset conduction abnormalities after TAVR, providing reference value for clinical doctors to better prevent and treat conduction abnormalities.

**Methods:**

Patients who underwent TAVR were divided into those who developed heart block and those who did not. Baseline clinical characteristics, cardiac structural parameters, procedural characteristics, electrocardiogram (ECG) changes before and after TAVR (△ = postoperative minus preoperative), and surgical complications were compared. Logistic regression was applied to identify significant risk factors for new-onset heart block.

**Results:**

We studied 93 patients, of whom 34.4% developed heart blocks. Univariate logistic regression showed that prior history of malignancy, atrial fibrillation, preoperative high-level total cholesterol and low-density lipoprotein cholesterol (LDL-C), △HR, △QRS interval, △QT interval, and △QTc interval were risk factors of new-onset heart block after TAVR. Multivariate analysis showed that preoperative high-level LDL-C and △QRS interval remained significant independent risk factors after adjusting for potential confounds.

**Conclusions:**

Heart block is the most common complication of TAVR, and its significant independent risk factors include high-level LDL-C and △QRS interval.

## 1. Introduction

Aortic stenosis (AS) is one of the common cardiac valvular diseases in elderly patients. It is characterized by progressive valve stenosis. Due to the aging of the population, the prevalence rate is expected to double in the next 20 years. The survival period of patients with severe AS is greatly shortened and the mortality rate is very high [1, 2, 3]. Therefore, it is necessary to replace the aortic valve in time. However, most elderly patients are weak, have poor tolerance to surgical aortic valve replacement, and have high surgical risk. Minimally invasive transcatheter aortic valve replacement (TAVR) has become a viable alternative in such patients [3, 4, 5]. TAVR can improve the clinical outcome of these patients, but the related clinical complications are relatively serious and complex. Conduction abnormalities are among the common complications of TAVR, which may tend to limit the promotion of this surgery to younger, lower-surgical-risk, populations [6]. Left bundle branch block (LBBB) is the most common type of heart block after TAVR, and its incidence in the first-generation valves is 4–65% [7, 8, 9, 10]. Of the different types of conduction abnormalities, high-grade atrioventricular block (HAVB) is among the more serious, with an incidence of 10–25% [11]. Most of those patients need to receive an implanted permanent pacemaker (PPM). In recent years, the precision of surgical instruments has been improved and data from surgeries have accumulated, but the incidence of conduction abnormalities has not decreased [12, 13, 14]. Conduction abnormalities may lead to further deterioration of cardiac function in patients with AS, increase the risk of heart failure (HF) and death, and adversely affect prognosis. Accordingly, predicting, preventing, and treating heart block has become the next frontier for improving TAVR outcomes. The present study analyzed the risk factors of new-onset heart block after TAVR in patients with severe AS and HF.

## 2. Methods

### 2.1 Study Population

Our study complied with the Declaration of Helsinki ethics statement. The study received approval from the institutional scientific review board. All patients provided written informed consent. The study sample included patients who underwent TAVR at the Yantai Yuhuangding Hospital Affiliated to Qingdao University and the affiliated Second Hospital of Tianjin Medical University, from January 2017 to September 2022. The inclusion criteria included: (1) underwent TAVR for symptomatic severe AS (mean gradient ≥40 mmHg [1 mmHg = 0.133 kPa], peak velocity ≥4.0 m/s, valve area ≤1 ${\mathrm{cm}}^{2}$ [or ≤0.6 ${\mathrm{cm}}^{2}$${\mathrm{/m}}^{2}$]); (2) a diagnosis of HF [15]; (3) surgical indication for TAVR. The exclusion criteria were: (1) mild or moderate AS; (2) could not be measured by echocardiography; (3) complete atrioventricular block or had pacemaker implantation prior to the TAVR; and (4) without HF. According to whether heart block occured after TAVR, patients were divided into heart block group and no heart block group.

### 2.2 Pre-TAVR Assessment

All patients were assessed by the TAVR cardiac team. The indication for TAVR, procedural concerns, surgical access site, as well as transcatheter heart valve type and size, were discussed and determined based on preoperative imaging examinations that included echocardiography, multislice computed tomography (MSCT), and angiography.

### 2.3 TAVR Procedure

All patients received general anesthesia. The main procedures included: (1) selection of the appropriate valve size according to the preoperative examination results; (2) femoral artery approach was the first choice (5 cases of transapical TAVR), temporary pacemaker was placed, pigtail catheter was placed to the base of the non-coronary sinus, and aortic root angiography was performed to assist in positioning; (3) the transmitter was passed to the aortic root, followed by release of the valve under the guidance of rapid pacing and aortic root angiography. Different release strategies were adopted according to different situations with the goal of fitting the stent valve to the valve ring; (4) aortography was performed and the patient was monitored for possible perivalvular leakage or obstruction of the coronary artery orifice.

### 2.4 Data Collection and Analysis

Baseline characteristics for each participant were collected. These included demographics (age, sex), New York heart association (NYHA) class, smoking, drinking, medical comorbidities such as hyperlipidemia, diabetes, dyslipidemia, malignant tumor, coronary heart disease, atrial fibrillation (AF), and previous history of cardiac surgery. Laboratory analyses included B-type natriuretic peptide (BNP), uric acid, total glyceride, total cholesterol (TC), low-density lipoprotein cholesterol (LDL-C) and high-density lipoprotein cholesterol, creatinine, and glomerular filtration rate. All echocardiograms were obtained with the patient in a stable hemodynamic condition. Echocardiographic parameters included left atrium anteroposterior diameter, left ventricular end-diastolic dimension, right ventricular anteroposterior diameter, etc. We also collected procedural characteristics, such as surgical approach, valve oversize rate, valve type, and valve size. Valve oversize rate = (valve model/aortic annulus diameter-1) × 100%. The diameter of the aortic annulus was obtained from computed tomography (CT)-evaluation results. We also collected preoperative electrocardiogram (ECG) and immediate postoperative electrocardiogram, and recorded the occurrence of heart block within 1 month after TAVR. Electrocardiogram parameters included heart rate (HR), QRS time course, QT interval, and QTc interval. The △QRS, △QT, and △QTc were obtained by postoperative values minus preoperative values. The adverse events studied were vascular complications, heart block, AF, poor wound healing, secondary thoracotomy for hemostasis, coronary obstruction, perivalvular leakage, need for permanent pacemaker, stroke, and all-cause mortality. Adverse-events data were obtained from medical records or by inquiries of the patients’ families or referring physicians via telephone.

### 2.5 Statistical Analysis

Data analysis was performed with SPSS (version 25.0, IBM Corp., Armonk, NY, USA). First, we ran univariate analyses. Categorical variables were expressed as n (%). Categorical data were compared using the Chi-square test or Fisher’s exact probability test. The Shapiro-Wilk test is used to analyze the normality of the continuous data. Normally distributed continuous variables were presented as mean (standard deviation, SD). Non-normally distributed continuous variables are presented as median (interquartile range, IQR). Continuous data were compared using Student’s *t*-test (normality) or Mann-Whitney U test (non-normality). Parameters with *p* ≤ 0.05 in univariate analyses were tested in multivariate analyses. A *p*-value < 0.05 in multivariate analysis was considered statistical significance.

## 3. Results

### 3.1 Occurrence of Heart Block after TAVR

A total of 93 patients were included, of whom 32 developed new-onset heart block after TAVR. Among those 32, 3 presented with first-degree atrioventricular block (2 of these had concurrent LBBB), which occurred within 24 h after TAVR. There were 9 patients who presented with third-degree atrioventricular block, of which 8 occurred within 24 h and 1 occurred within 10 days after TAVR. There were 19 patients who presented with LBBB, of which 16 occurred within 24 h after the operation. Six of those 16 cases (37.5%) recovered during follow-up. Three cases showed a delayed presentation. Right bundle branch block (RBBB) occurred in all 3 cases, of which 2 occurred within 24 h (one of 2 cases [50.0%] recovered during follow-up). One case occurred within 3 days after TAVR. Pacemakers were implanted in 9 patients.

### 3.2 Comparison of Clinical Characteristics

There were no statistical differences in baseline characteristics between the two groups, except for prior malignancy and AF, which were of significantly higher frequency in patients who subsequently developed heart block (malignancy: 18.8% vs. 3.3%, *p* = 0.032; AF: 34.4% vs. 8.2%, *p* = 0.001). Patients who developed new-onset heart block were more likely to have higher TC (4.87 [1.15] vs. 4.34 [1.13], *p* = 0.038) and LDL-C (3.03 [0.78] vs. 2.57 [0.94], *p* = 0.020) before TAVR. As for the echocardiography parameters, there were no statistically significant differences between the two groups (Table 1).

**Table 1.** **Comparison of clinical characteristics between 2 groups**.

	Variables		Heart block (n = 32)	No heart block (n = 61)	*T*, *Z* or $\chi^{2}$	*p*
Baseline data	Age (years), Mean (SD)		75.03 (6.41)	72.28 (7.62)	1.743	0.085
	BMI (${\mathrm{kg/m}}^{2}$), Mean (SD)		23.94 (3.85)	24.50 (3.20)	0.747	0.457
	Women, n (%)		17 (53.1%)	27 (44.3%)	0.661	0.416
	NYHA class, n (%)				4.232	${\mathrm{0.221}}^{\Delta }$
		I	4 (12.5%)	2 (3.3%)		
		II	2 (6.3%)	3 (4.9%)		
		III	14 (43.8%)	37 (60.7%)		
		IV	12 (37.5%)	19 (31.1%)		
	Smoking, n (%)		5 (15.6%)	21 (34.4%)	3.684	0.055
	Drinking, n (%)		3 (9.4%)	14 (23.0%)	2.590	0.108
	Hypertension, n (%)		21 (65.6%)	35 (57.4%)	0.596	0.440
	Diabetes, n (%)		9 (28.1%)	17 (27.9%)	0.001	0.979
	Tumor, n (%)		6 (18.8%)	2 (3.3%)	4.574	**0.032**
	CHD, n (%)		15 (46.9%)	35 (57.4%)	0.931	0.335
	Prior MI, n (%)		3 (9.4%)	7 (11.5%)	0.000	1.000
	AF, n (%)		11 (34.4%)	5 (8.2%)	10.098	**0.001**
	Sinus bradycardia, n (%)		1 (3.1%)	7 (11.5%)	0.951	0.329
	First-degree AVB, n (%)		5 (15.6%)	15 (24.6%)	0.999	0.317
	LBBB, n (%)		0 (0.0%)	5 (8.2%)	1.395	0.238
	RBBB, n (%)		3 (9.4%)	3 (4.9%)	0.150	0.699
	Cerebral infarction, n (%)		5 (15.6%)	7 (11.5%)	0.058	0.809
	CABG, n (%)		1 (3.1%)	0 (0.0%)		${\mathrm{0.344}}^{\Delta }$
	PCI, n (%)		5 (15.6%)	11 (18.0%)	0.085	0.770
Laboratory examination	TG (mmol/L), Median (IQR)		0.93 (0.78, 1.31)	1.01 (0.77, 1.36)	0.724	0.469
	TC (mmol/L), Mean (SD)		4.87 (1.15)	4.34 (1.13)	2.100	**0.038**
	LDL-C (mmol/L), Mean (SD)		3.03 (0.78)	2.57 (0.94)	2.373	**0.020**
	HDL-C (mmol/L), Median (IQR)		1.24 (0.99, 1.51)	1.24 (0.96, 1.43)	0.501	0.616
	BNP (pg/mL), Median (IQR)		939.13 (224.70, 2770.12)	577.87 (179.10, 1461.01)	1.059	0.290
	Ccr (mL/min), Mean (SD)		71.63 (26.21)	72.87 (26.68)	0.214	0.831
	Uric acid (µmol/L), Median (IQR)		415.00 (271.50, 483.75)	411.00 (300.00, 515.00)	0.481	0.630
Echocardiography	AO ascending segment (mm), Median (IQR)		38.00 (32.00, 41.00)	34.00(31.90, 39.00)	1.691	0.091
	LAAD (mm), Mean (SD)		44.09 (5.97)	43.94 (5.51)	0.125	0.901
	LVEDD (mm), Mean (SD)		49.30 (7.51)	51.55 (7.34)	1.391	0.168
	RVAD (mm), Median (IQR)		23.95 (21.83, 26.00)	23.00 (21.00, 24.35)	1.463	0.143
	LVEF (%), Median (IQR)		60.00 (52.50, 65.75)	57.00 (44.00, 64.00)	1.554	0.120
	PASP >60 mmHg, n (%)		4 (12.5%)	18 (29.5%)	3.362	0.067

Remarks: Values are presented as mean (SD), median (IQR) or n (%). *p* values ≤ 0.05 in bold. BMI, body mass index; NYHA, New York heart association; CHD, coronary heart disease; MI, myocardial infarction; AF, atrial fibrillation; AVB, atrioventricular block; LBBB, left bundle branch block; RBBB, right bundle branch block; CABG, coronary artery bypass surgery; PCI, percutaneous coronary intervention; TG, total glyceride; TC, total cholesterol; LDL-C, low-density lipoprotein cholesterol; HDL-C, high-density lipoprotein cholesterol; BNP, B-type natriuretic peptide; Ccr, creatinine clearance rate; AO, aorta; LAAD, left atrium anteroposterior diameter; LVEDD, left ventricular end-diastolic dimension; RVAD, right ventricular anteroposterior diameter; LVEF, left ventricular ejection fraction; PASP, pulmonary artery systolic pressure; SD, standard deviation; IQR, interquartile range. Δ Fisher’s exact tests.

### 3.3 Comparison of Surgical Parameters

There was no significant difference between the heart block group and the no heart block group in terms of bicuspid aortic valve, surgical approach, balloon size, pre-dilation and post-dilation, valve oversize rate, valve-in-valve surgery, valve type and size (Table 2).

**Table 2.** **Comparison of surgical parameters between 2 groups**.

Variables		Heart block (n = 32)	No heart block (n = 61)	*Z or χ^2^*	*p*
BAV, n (%)		11 (34.4%)	18 (29.5%)	0.232	0.630
TAVR access, n (%)				1.395	0.238
	TF-TAVR	32 (100.0%)	56 (91.8%)		
	TA-TAVR	0 (0.0%)	5 (8.2%)		
Pre-dilated balloon size (mm), Median (IQR)		22.00 (18.00, 23.00)	22.00 (20.00, 23.00)	1.807	0.071
Post-dilated balloon size (mm), Median (IQR)		22.00 (20.00, 25.00)	22.00 (20.00, 23.75)	0.257	0.797
Pre-dilation, n (%)				3.504	${\mathrm{0.326}}^{\Delta }$
	0	6 (18.8%)	6 (9.8%)		
	1	26 (81.3%)	50 (82.0%)		
	2	0 (0.0%)	4 (6.6%)		
	3	0 (0.0%)	1 (1.6%)		
Post-dilation, n (%)				3.745	${\mathrm{0.109}}^{\Delta }$
	0	22 (68.8%)	34 (55.7%)		
	1	9 (28.1%)	27 (44.3%)		
	2	1 (3.1%)	0 (0.0%)		
Valve oversize rate (%), Median (IQR)		9.40 (3.25, 14.85)	8.21 (0.44, 15.33)	0.311	0.755
Valve-in-valve surgery, n (%)		3 (9.4%)	7 (11.5%)	0.000	1.000
Valve type, n (%)				4.829	${\mathrm{0.069}}^{\Delta }$
	J-VALVE	0 (0.0%)	5 (8.2%)		
	Vita Flow	4 (12.5%)	15 (24.6%)		
	VENUS A	28 (87.5%)	41 (67.2%)		
Valve size (mm), Median (IQR)		26.00 (23.25, 29.00)	26.00 (23.00, 29.00)	0.671	0.503

Remarks: Values are presented as median (IQR) or n (%). BAV, bicuspid aortic valve; TAVR, transcatheter aortic valve replacement; TF-TAVR, transfemoral TAVR; TA-TAVR, transapical TAVR; IQR, interquartile range; J-VALVE, VENUS A, and Vita Flow are the names of valves. Δ Fisher’s exact tests.

### 3.4 ECG before and after TAVR

The median difference between postoperative and preoperative heart rate (△HR) was higher in the heart block group (*p* < 0.01). Similarly, the medians of the differences between postoperative and preoperative QRS time course (△QRS), QT interval (△QT), and QTc interval (△QTc), were longer in the heart block group (*p* < 0.001) (Table 3).

**Table 3.** **Comparison of ECG changes before and after operation in 2 groups of patients**.

Variable	Heart block (n = 32)	No heart block (n = 61)	*T or Z*	*p*
HR (BPM), Median (IQR)	74.50 (65.50, 90.75)	70.00 (62.00, 80.50)	1.740	0.082
PR interval (ms), Median (IQR)	179.50 (151.75, 199.25)	181.00 (161.50, 204.75)	0.304	0.761
QRS wave (ms), Median (IQR)	96.50 (91.00, 109.50)	102.00 (91.50, 115.50)	1.185	0.236
QT interval (ms), Mean (SD)	397.94 (55.85)	419.37 (49.78)	1.884	0.063
QTc interval (ms), Median (IQR)	451.00 (429.50, 470.25)	460.00 (428.00, 483.50)	1.096	0.273
△HR (BPM), Median (IQR)	–9.50 (–20.00, 4.00)	2.00 (–8.00, 11.00)	2.815	**0.005**
△QRS wave (ms), Median (IQR)	52.00 (24.25, 71.00)	2.00 (–6.00, 10.00)	6.019	< **0.001**
△QT interval (ms), Median (IQR)	77.50 (53.25, 108.00)	16.00 (–34.50, 52.00)	4.117	< **0.001**
△QTc interval (ms), Median (IQR)	70.50 (39.50, 94.75)	10.00 (–22.00, 48.50)	4.149	< **0.001**

Remarks: Values are presented as mean (SD) or median (IQR). *p* values ≤ 0.05 in bold. ECG, electrocardiogram; BPM, beat per minute; SD, standard deviation; IQR, interquartile range; HR, heart rate. △HR, △QRS wave, △QT interval and △QTc interval are all obtained by subtracting preoperative parameters from postoperative parameters.

### 3.5 Analysis of Independent Risk Factors of Heart Block after TAVR

Univariate analysis was used to identify significant risk factors of new-onset heart block after TAVR (Tables 1,2,3). Variables with *p*-values ≤ 0.05 from either intergroup comparison or on logistic regression were entered into multivariable logistic regression models. High preoperative LDL-C (OR 2.042, 95% CI [1.072, 3.892], *p* = 0.030) and △QRS duration (OR 1.056, 95% CI [1.033, 1.079], *p* < 0.001) were independent risk factors for heart block after TAVR (Table 4).

**Table 4.** **Multivariable logistic regression analysis of heart block after TAVR**.

Variable	Multifactor Logistic Regression Analysis			
	β	OR	95% CI	*p*
LDL-C (mmol/L)	0.714	2.042	(1.072, 3.892)	**0.030**
△QRS (ms)	0.054	1.056	(1.033, 1.079)	< **0.001**

Remarks: *p* values ≤ 0.05 in bold. TAVR, transcatheter aortic valve replacement; OR, odd ratio; CI, confidence interval; LDL-C, low-density lipoprotein cholesterol. △QRS wave is obtained by subtracting preoperative QRS wave from postoperative QRS wave.

### 3.6 Short-Term Postoperative Complications 

Regarding short-term postoperative complications in the heart block group, there was one case of vascular complications, one case of cardiac perforation, and two cases of moderate perivalvular leakage. In the group without heart block, there was one case of acute kidney injury, three cases of vascular complications, one case of poor wound healing, two cases of moderate perivalvular leakage, and four cases of cerebrovascular diseases. However, there was no significant statistical difference between groups in complication rate.

## 4. Discussion

The main findings of our study are as follows: (a) the prevalence of new-onset heart block was 34.4%, and permanent pacemaker implantation (PPI) was performed in 9.7% of the cases; (b) patients who developed new-onset heart block were more likely to have malignancy and AF, as well as higher levels of TC and LDL-C before TAVR; (c) multivariate logistic regression showed that high preoperative LDL-C and △QRS duration were important independent risk factors for heart block after TAVR.

Different complications can occur after TAVR, of which many have been identified as independent predictors of mortality [16, 17]. New-onset LBBB is one of the most common complications post-TAVR. At times, transient new-onset LBBB persists at discharge or by 30 days afterward in approximately 55% of cases, leading to a PPI in 10–20% of cases, most often for HAVB [8, 18, 19]. Previous studies have reported that new LBBB was associated with increased risks of late (≥1 year after TAVR) all-cause mortality and HF hospitalizations [18]. Chamandi *et al*. [20] found that LBBB had no impact on long-term mortality or HF hospitalization post-TAVR, but did increase the risk of PPI and non-improvement in LVEF over time. Another 5-year follow-up study showed that new LBBB was associated with increased risk of HF hospitalizations [21]. The presence of HAVB after TAVR usually indicates deterioration of cardiac function and increased risk of death. Therefore, in the present study we sought to define the rates of heart block and identify risk factors of its development in this cohort. Previous studies have identified the following risk factors: male sex, aortic valve calcification (AVC), diabetes mellitus, AF, and surgical operation factors (pre-existing conduction abnormalities, valve type, larger prosthesis size, valve oversizing, and increasing implantation depth) [22, 23, 24, 25, 26]. Our study was not able to conclude that preoperative conduction abnormalities, valve type, valve size, surgical approach, and pre-dilation, are risk factors for new postoperative conduction abnormalities; this may be due to the small sample size. However, our research continues. We are collecting more TAVR cases to verify the currently recognized risk factors and to discover new risk factors for postoperative cardiac block, with a view to improving the prognosis of patients. Due to the fact that this was a dual-center study, there are a few missing data on the depth of valve implantation. We conducted a statistical analysis of existing data, and the results showed that there was no statistical difference in valve implantation depth between the two groups. In the future, we will collect more TAVR cases and supplement this information to study the relationship between valve implantation depth and postoperative conduction block. In addition to the above factors, our research found that high levels of LDL-C and a greater postoperative-preoperative difference in QRS duration were independent risk factors of heart block. At least two possible mechanisms may explain the higher rates of heart block in patients who had higher baseline levels of LDL-C. (1) Elevated levels of LDL-C are the major culprit in the development of atherosclerosis [27]. The high blood viscosity and atherosclerotic coronary stenosis in patients with dyslipidemia may lead to myocardial ischemia and hypoxia, and then promote the occurrence of heart block. (2) Anatomically, the atrioventricular (AV) bundle penetrates the central fibrous body at the right fibrous trigone and continues at the membranous part of the interventricular septum located in the area under the right and the noncoronary aortic cusps, forming the left bundle branch [28]. Damage caused by direct mechanical interaction of the valve stent frame with the AV conduction system in the left ventricular outflow tract may lead to new-onset LBBB or complete AV block after TAVR. Previous studies have confirmed that an increased level of circulating oxidized low-density lipoprotein (ox-LDL) is associated with worse fibrocalcific remodeling of valvular tissue in AS [29, 30]. The plasma level of ox-LDL was significantly associated with LDL-C [30]. Therefore, we speculate that (a) The higher the level of LDL-C, the more serious the degree of AVC. During valve implantation, pre-dilation, post-dilation, and valve expansion, produce radial force on the conduction system near the aortic valve. Especially when the aortic valve is severely calcified, the expansion of the artificial valve will push the calcified mass of the autogenous aortic valve to the surrounding tissue, which will intensify the compression effect on the conduction system and cause the occurrence of heart block. In addition, the uneven deposition of lipids in the valve may cause asymmetric calcification, which may cause the expanded prosthesis to deviate from the centerline in the direction of the area under the right and noncoronary aortic cusps. The consequent mechanical stress on the AV conduction system might be increased locally due to the uneven distribution of radial forces, which may lead to an increase in the probability of heart block. (b) The deposit of lipid particles in the conduction system as a result of its proximity to the aortic valve complex can lead to conduction abnormalities. Currently, Lipoprotein(a) [Lp(a)] is a research hotspot. Early studies have confirmed that high plasma Lp(a) concentration is a risk factor for cardiovascular disease, that is, myocardial infarction (MI) and atherosclerotic stenosis [31, 32, 33]. It is also a risk factor for postoperative cardiovascular and cerebrovascular adverse events in patients with acute MI undergoing percutaneous coronary intervention [34]. Recent studies have shown that Lp(a) is an important risk factor for incident AS [35, 36]. And it is also a component of LDL-C [37]. The statistical results of most of our data showed the proportion of patients with elevated Lp(a) levels in the heart block group is relatively high, but there is no statistically significant difference in preoperative Lp(a) levels between the two groups. We consider that this may be related to a small sample size. In future studies, we will include more research subjects, collect Lp(a) data, and further investigate whether it is a contributing factor to LDL-C-induced heart block. The △QRS duration was also a risk factor, suggesting that overall intraventricular conduction delays, which may be caused by damage to the left bundle branch, lead to LBBB, which in turn increases the probability of developing HAVB [38].

Altogether, these results suggest that prevention strategies such as smoking cessation, better control of blood pressure, and LDL-C reduction, can delay the progression of AS and associated conduction abnormalities, possibly by reducing inflammation [39, 40]. The impact of TAVR on the conduction system is dynamic; both the timing and duration of conduction block are uncertain, which adds difficulty to the management of related conduction abnormalities. Pacing is the most effective intervention for the newly emerged conduction block. However, the ideal timing of pacing therapy remains uncertain. The determination of the pacing treatment is usually based on the clinical judgment. It should be noted that conduction abnormalities can show delayed presentations, which may be related to tissue edema and late expansion of the prosthesis. If HAVB occurs after TAVR, a PPM is implanted. For high-risk LBBB patients, ECG monitoring can be extended to at least 2–4 weeks. If necessary, further electrophysiological examination can be performed [9]. After TAVR, if dynamic ECG monitoring is not available, ECG monitoring should be conducted regularly, especially focusing on the change of QRS duration, so as to identify high-risk patients with HAVB.

In the present study, several patients had received 24-h ECG monitoring before TAVR. Some were found to have previously unknown paroxysmal arrhythmia or transient conduction disorder, most of whom were asymptomatic. Thus, many baseline arrhythmic events in TAVR patients go undetected or unrecognized. This may lead to bias whereby the rates of new-onset heart block are overestimated. Due to its potential clinical benefits and relatively low cost, the recommendation is for ECG monitoring for at least 24 h before TAVR. For patients with newly discovered arrhythmia, appropriate therapy should be carried out promptly in accordance with the recommendations of current guidelines. The application of 24-h ECG may provide an opportunity to determine the actual incidence of tachyarrhythmias and bradyarrhythmias attributable to the transcatheter prosthesis and the TAVR procedure, which is substantial for the preoperational evaluation of the newer transcatheter valve systems or new indications of TAVR.

This study has some limitations that should be recognized. First, our study should be interpreted with caution due to relatively small sample size. Moreover, due to the routine joint analysis, there are a few missing surgical data. Second, some patients with severe AS did not have ECG monitoring for at least 24 h before surgery, which led to overestimation of abnormal conduction after TAVR. Finally, most patients for TAVR undergo only 12-lead ECG monitoring after TAVR so we inevitably lose some key information on intermittent conduction abnormalities.

## 5. Conclusions

Patients who developed new-onset heart block were more likely to have prior histories of malignancy and AF, and have higher levels of TC and LDL-C before TAVR. High preoperative LDL-C levels and higher △QRS duration were independent risk factors.

## Data Availability

The datasets used and analyzed during the current study are available from the corresponding author on reasonable request.
